# Production of D-Allose From D-Allulose Using Commercial Immobilized Glucose Isomerase

**DOI:** 10.3389/fbioe.2021.681253

**Published:** 2021-07-15

**Authors:** Mi Na Choi, Kyung-Chul Shin, Dae Wook Kim, Baek-Joong Kim, Chang-Su Park, Soo-Jin Yeom, Yeong-Su Kim

**Affiliations:** ^1^Wild Plants Industrialization Research Division, Baekdudaegan National Arboretum, Bonghwa, South Korea; ^2^Department of Integrative Bioscience and Biotechnology, Konkuk University, Seoul, South Korea; ^3^Starch and Sweetener Research Department, Ingredient R&D Center, DAESANG Corporation, Icheon, South Korea; ^4^Department of Food Science and Technology, Daegu Catholic University, Gyeongsan, South Korea; ^5^School of Biological Sciences and Technology, Chonnam National University, Gwangju, South Korea

**Keywords:** D-allose, D-allulose, rare sugar, packed bed reactor, Sweetzyme IT, glucose isomerase

## Abstract

Rare sugars are regarded as functional biological materials due to their potential applications as low-calorie sweeteners, antioxidants, nucleoside analogs, and immunosuppressants. D-Allose is a rare sugar that has attracted substantial attention in recent years, owing to its pharmaceutical activities, but it is still not widely available. To address this limitation, we continuously produced D-allose from D-allulose using a packed bed reactor with commercial glucose isomerase (Sweetzyme IT). The optimal conditions for D-allose production were determined to be pH 8.0 and 60°C, with 500 g/L D-allulose as a substrate at a dilution rate of 0.24/h. Using these optimum conditions, the commercial glucose isomerase produced an average of 150 g/L D-allose over 20 days, with a productivity of 36 g/L/h and a conversion yield of 30%. This is the first report of the successful continuous production of D-allose from D-allulose by commercial glucose isomerase using a packed bed reactor, which can potentially provide a continuous production system for industrial applications of D-allose.

## Introduction

A first-generation sweetener is a sweetness-oriented sugar such as sucrose, fructose, and glucose. Second-generation sweeteners are low-calorie and high-sweetness sugars, including the sugar alcohol xylitol, sucralose, aspartame, and oligosaccharides, which participate in intestinal regulation. Currently, functional rare sugars are drawing attention as third-generation sweeteners.

A rare sugar is defined as a monosaccharide that is rarely found in nature according to the International Society of Rare Sugars (ISRS) (Izumori, 2002, 2006). There are more than 50 kinds of rare monosaccharides. Two of these, D-tagatose and D-allulose, but not D-allose, have been formally approved by the United States Food and Drug Administration (FDA) as Generally Recognized As Safe (GRAS), and are allowed for use in the food industries (Levin, 2002; Kim, 2004; Mu et al., 2012).

The rare sugarD-allose has attracted substantial attention in recent years because of its beneficial biological properties as an anti-cancer (Sui et al.,2005a,b; Mitani et al., 2009; Kanaji et al., 2018), anti-oxidant (Murata et al., 2003), anti-inflammatory (Gao et al., 2001), and anti-hypertensive (Kimura et al., 2005) agent, and its ability to protect against ischemia-reperfusion injury of the liver (Hossain et al., 2003). Additionally, D-allose is a non-caloric and non-toxic sweetener that has approximately 80% the sweetness of sucrose (Chattopadhyay et al., 2014; Mooradian et al., 2017; Chen et al., 2018). Despite these various benefits, D-allose occupies a small proportion of the industrial market due to its scarcity and high production costs. Thus, the continuous production of D-allose is important for broadening its industrial applications. However, only the production of D-allose using free enzyme has been studied until now. For the industrial production of D-allose, which is an important next-generation sweetener, it is necessary to study the application of a reactor using a food grade commercial enzyme.

D-Allose can be synthesized by chemical methods (Bernaerts et al., 1963; Baker et al., 1972) but some disadvantages exist from these approaches, such as complicated purification steps, undesirable by-products, environmental pollution from chemical waste, low productivity, and the inability to reuse substrates (Lim and Oh, 2011). For these reasons, the biological synthesis of D-allose is becoming a key foundation. Biologically,D-allose can be synthesized from D-glucose by a three-step enzyme-catalyzed pathway. In the first reaction, D-glucose is converted to D-fructose by D-glucose isomerase. The second reaction, the conversion of D-fructose to D-allulose, can be catalyzed by D-tagatose 3-epimerase or D-allulose 3-epimerase. D-Allose is produced in the final step by the conversion of D-allulose by L-rhamnose isomerase from *Clostridium stercorarium* (Seo et al., 2018), *Thermobacillus composti* (Xu et al., 2017), *Bacillus subtilis* (Bai et al., 2015), or *Pseudomonas* sp. (Bhuiyan et al., 1998); by ribose-5-phosphate isomerase from *Clostridium thermocellum* (Park et al., 2007b) or *Thermotoga lettingae* (Feng et al., 2013); or by galactose 6-phosphate isomerase from *Lactococcus lactis* (Park et al., 2007a). Recently, a one-pot reaction method was reported to successfully produce D-allose from D-fructose using D-allulose 3-epimerase from *Flavonifractor plautii* and D-ribose-5-phosphate isomerase from *C. thermocellum* (Lee et al., 2018).

Here, we describe the first demonstration of the continuous production of D-allose from D-allulose using commercial food-grade D-glucose isomerase (Sweetzyme IT; GI) in a packed bed reactor.

## Materials and Methods

### Materials

Food-grade immobilized GI from *Streptomyces murinus* (Sweetzyme IT) was purchased from Novozyme (Kalubdborg, Denmark). D-allose, D-allulose, and other reagents were purchased from Sigma Aldrich (St. Louis, MO, United States). The packed bed reactor XK26/100 was purchased from GE Healthcare Life Science (Uppsala, Sweden).

### Enzyme Assay

The activity of GI was measured in a reaction mixture containing 50 mM EPPS buffer (pH 8.0), 10 g/L D-allulose, and 10 mg/mL GI at 60°C for 30 min. One unit of enzyme activity was defined as the amount of enzyme required to produce 1 μmol of D-allose from D-allulose per minute at 60°C and pH 8.0.

### Optimization of D-Allose Production Conditions

To determine the optimum pH and temperature for the GI-catalyzed production of allose, the reactions were performed for 30 min by varying the buffer pH from pH 4–9 at 60°C and by varying the temperature from 40–90°C at pH 8.0 using 10 g/LD-allulose as a substrate. The thermostability of GI was monitored as a function of time for incubation by incubating the enzyme solution at different temperatures (50–90°C) in 50 mM EPPS buffer (pH 8.0). Samples were withdrawn at various time intervals and then assayed in 50 mM EPPS buffer (pH 8.0) containing 10 g/L D-allulose at 70°C for 30 min.

### Continuous D-Allose Production

The dilution rate and substrate concentration for continuous D-allose production in the packed bed reactor were investigated. The immobilized GI was packed into a XK26/100 packed bed reactor with a bed volume of 300 mL and the D-allulose concentration was varied from 100 to 700 g/L with a dilution rate of 0.24/h for 5 h. The optimum dilution rate was determined by varying the rate from 0.07 to 0.95/h using a 500 g/L D-allulose solution and a Gilson Mini Plus evolution peristaltic pump (Gilson, Inc., WI, United States) at 60°C. The conversion yield was calculated as the percentage of the concentration of the produced D-allose as a product in relation to the concentration of D-allulose as a substrate put in the reaction.

### Analytical Methods

D-Allulose and D-allose concentrations were determined using a Bio-LC system (Dionex ICS-3000, Sunnyvale, CA, United States) with an electrochemical detector and a CarboPac PAI column. The column was eluted at 30°C with 0.1 M NaOH (0–5 min), followed by a linear gradient of sodium acetate (0–0.2 M) at 1 mL/min for 5–35 min.

### Nuclear Magnetic Resonance Analysis

The nuclear magnetic resonance (NMR) spectra of D-allose were recorded on a Bruker 800 MHz spectrometer (Bruker, Karlsruhe, Germany) using standard Bruker pulse programs. Chemical shifts were given as δ-values with reference to tetramethylsilane (TMS) as an internal standard. ^1^H and ^13^C-NMR assignments were determined by gHSQC, gHMBC, and ^1^H-^1^H-COSY.

## Results and Discussion

### D-Allose Production From D-Allulose Using a Commercial Enzyme

Previously, D-allose has been produced using microbial enzymes, including L-rhamnose isomerase (Seo et al., 2018), D-ribose-5-phosphate isomerase (Feng et al., 2013), D-galactose-6-phosphate isomerase (Park et al., 2007a), and D-glucose-6-phosphate isomerase (Yoon et al., 2009). The most effective enzyme reported to date was L-rhamnose isomerase (Lim and Oh, 2011; Mu et al., 2012; Chen et al., 2018). The conserved protein domain family of L-rhamnose isomerase belongs to the AP2Ec super-family; this family includes other sugar-converting enzymes such as xylose (D-glucose) isomerase. The substrate specificities of commercial glucose isomerase from *S. murinus* (Sweetzyme IT, GI) and L-rhamnose isomerase from *Dictyoglomus turgidum* have also been reported for L-rhamnose and D-fructose (Kim et al., 2013, 2018).

**FIGURE 1:**
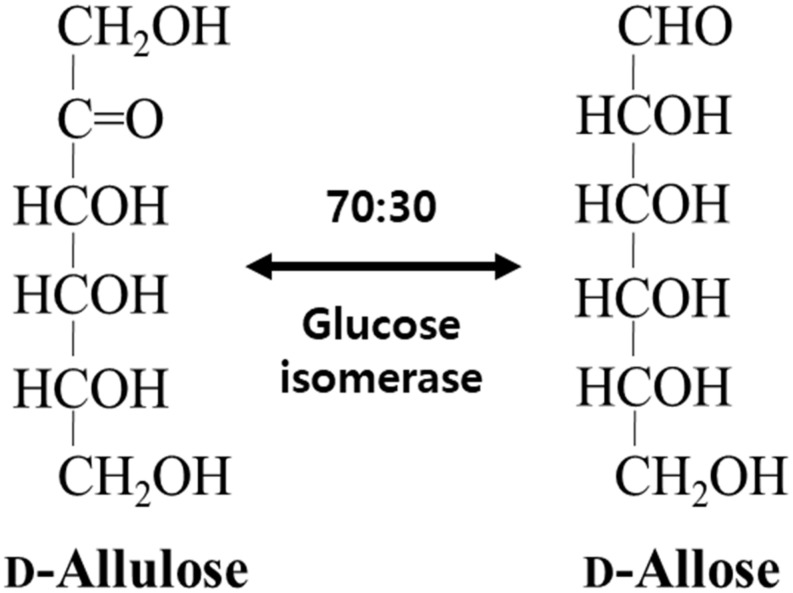
Schematic diagram for the isomerization of D-allulose and D-allose catalyzed by commercial glucose isomerase.

Not only does GI catalyze the reversible isomerization of D-glucose and D-xylose to D-fructose and D-xylulose, respectively, but the isomerization of allose was catalyzed by a recombinant GI from *Streptomyces* sp. (Brucher et al., 2018) and a partially purified D-xylose isomerase from *Streptomyces albus* (Sanchez and Smiley, 1975). In this study, GI converted D-allulose to D-allose, and the specific activity of GI for D-allulose was 2.4- and 2.0-fold lower than those for D-glucose and D-xylose, respectively. Nevertheless, activity of GI toward D-allose was approximately 624-fold higher than that of a partially purified D-xylose isomerase from *S. albus* (Table 1). Thus, we successfully produced D-allose using D-allulose as a substrate by immobilized GI (see the reaction scheme in Figure 1). The reaction product was identified as a D-allose by NMR spectroscopy (Supplementary Figures 1–5). Commercial GI was used in this case because this enzyme provides enhanced reaction stability and better process control with minimization of the pressure drop issue by a large particle size of 0.4–1.0 mm, and it can be applied to the industrial production of D-allose (Allen et al., 1979). Using our system, the conversion yield of D-allulose to D-allose catalyzed by GI was approximately 30%. Previously reported conversion yields ofD-allose from D-allulose using microbial enzymes such as L-rhamnose isomerases from *C. stercorarium* (Seo et al., 2018), *T. composti* KWC4 (Xu et al., 2017), *B. subtilis* WB600 (Bai et al., 2015), *Caldicellulosiruptor saccharolyticus* (Lin et al., 2011), *Thermoanaerobacterium saccharolytium* NTOU1 (Lin et al., 2010), *Bacillus pallidus* Y25 (Poonperm et al., 2007), and *Pseudomonas stutzeri* (Morimoto et al., 2006) were 33, 23, 37.5, 33, 34, 35, and 25%, respectively. The yields using D-ribose 5-phosphate isomerases from *C. thermocellum* (Park et al., 2007b) and *T. lettingae* TMO (Feng et al., 2013) were 33% and 32%, respectively, and those using D-galactose-6-phosphate isomerase from *L. lactis* (Park et al., 2007a) and D-glucose-6-phosphate isomerase from *Pyrococcus furiosus* (Yoon et al., 2009) were 28, 25, and 32%, respectively. This difference in conversion yields is expected to be due to reaction environmental factors such as temperature, and pH, and type of reactor, because the equilibrium between D-allulose and D-allose is constant. To date, there are no studies using a reactor to produce D-allose from D-allulose. Although GI showed a lower conversion yield than many enzymes, it was applied to a packed bed reactor in this study because it is easy to industrialize as a commercially available immobilized enzyme.

**TABLE 1:** Specific activity of GI for sugars.

Substrate	Product	Specific activity (μmol/min/mg)
D-Glucose	D-Fructose	367 ± 32
D-Xylose	D-Xylulose	316 ± 15
D-Allulose	D-Allose	156 ± 33
D-Ribose	D-Ribulose	87 ± 7
L-Rhamnose	L-Rhamnulose	103 ± 11
D-Galactose	D-Tagatose	ND

*ND, not detected.*

### Effect of pH and Temperature on D-Allose Production

**FIGURE 2:**
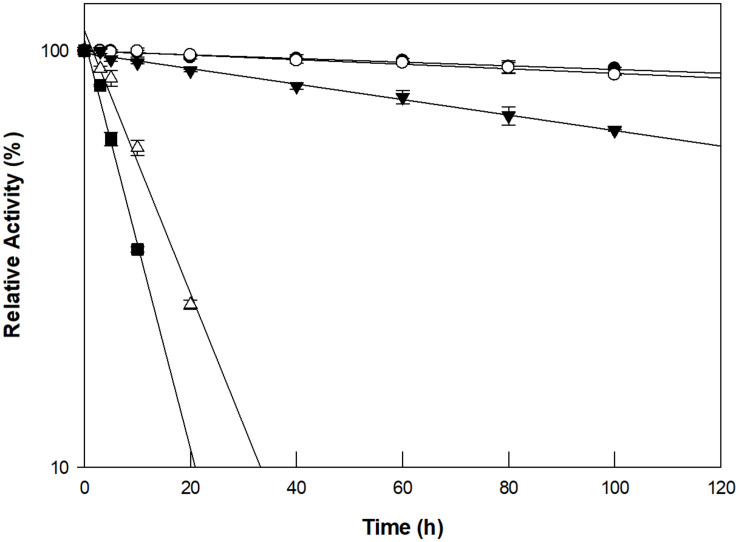
Thermal inactivation of GI catalyzing D-allose production at 50°C (closed circles), 60°C (open circles), 70°C (closed inverted triangles), 80°C (open triangles), and 90°C (closed squares). The enzyme was incubated at temperatures ranging from 50 to 90°C for varying periods of time. A sample was withdrawn at each time interval and assayed for enzyme activity in 50 mM EPPS buffer (pH 8.0) containing 10 g/L D-allulose at 60°C for 30 min. The experimental data for thermal deactivation of the enzyme were fitted to a first-order curve and the half-life of the enzyme was calculated using Sigma plot 10.0 software (Systat software, San Jose, CA, United States). Data represent the means of three experiments ± standard deviation (SD).

The optimum pH and temperature for D-allose production using GI were investigated using a pH range of 4–9 and a temperature range of 40–90°C. Maximum GI activity was observed at pH 8.0 and 90°C (Supplementary Figure 6). However, the thermostability of GI at 90°C could not sustain the production of D-allose (Figure 2). The half-life of GI activity at 50, 60, 70, 80, and 90°C was 1,021, 854, 352, 47, and 17 h, respectively. As a result, the optimum pH and temperature for the GI-catalyzed production of D-allose were determined to be pH 8.0 and 60°C, respectively. For the GI-catalyzed production of D-fructose from D-glucose and of L-rhamnulose from L-rhamnose, the optimum conditions were reported to be a pH 8.0–8.5 and 60–70°C (Kim et al., 2018). The optimal conditions for the production of D-allose using L-rhamnose isomerases from *C. stercorarium* (Seo et al., 2018), *T. composti* KWC4 (Xu et al., 2017), *B. subtilis* WB600 (Bai et al., 2015), immobilized L-rhamnose isomerase from *P. stutzeri* (Morimoto et al., 2006), D-ribose-5-phosphate isomerase from *C. thermocellum* (Park et al., 2007b), D-galactose-6-phosphate isomerase from *L. lactis* (Park et al., 2007a), and D-glucose-6-phosphate isomerase from *P. furiosus* (Yoon et al., 2009) were pH 7.0–9.0 and 30–75°C.

### Optimization of Reaction Conditions for Continuous D-Allose Production

**FIGURE 3:**
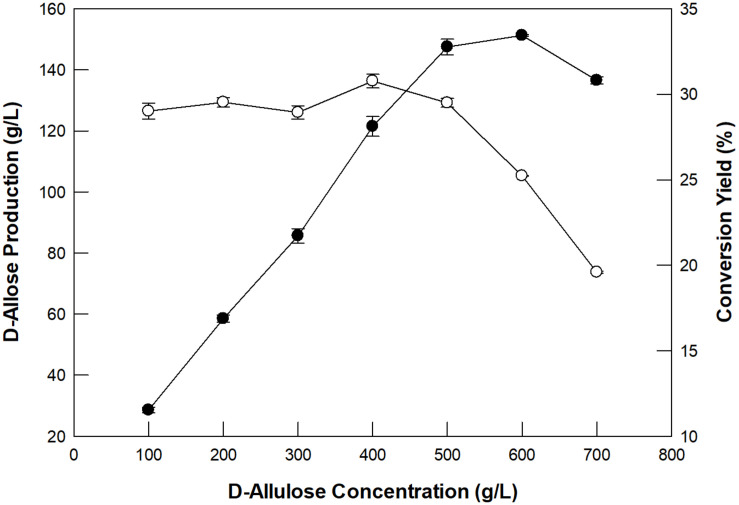
Effect of substrate concentration on D-allose production (closed circles) and conversion yield (open circles) in a packed bed reactor. The reactions were performed in 50 mM EPPS buffer (pH 8.0) containing 100–700 g/L D-allulose at 60°C at a dilution rate of 0.24/h. Data represent the means of three experiments ± SD.

The concentration ofD-allulose as a substrate and its dilution rate for the production of D-allose using GI were investigated in a packed bed reactor (XK26/100; i.d. 26 mm × length 1,000 mm). The enzyme was applied to the packed bed reactor with a working volume of 300 mL. D-Allulose (100–700 g/L) was fed continuously into the packed bed reactor at 60°C with a dilution rate of 0.24/h. The optimum concentration of D-allulose was 500 g/L (Figure 3). The concentration of D-allose produced increased up to 600 g/L D-allulose; however, the conversion yield of D-allulose toD-allose decreased when the D-allulose concentration exceeded 500 g/L. This may have been due to substrate inhibition at high concentrations. The optimum concentration of D-allulose for D-allose production using immobilized L-rhamnose isomerase from *P. stutzeri* was reported to be 500 g/L (Morimoto et al., 2006) and that of L-rhamnose for L-rhamnulose production using GI was reported to be 300 g/L (Kim et al., 2018).

**FIGURE 4:**
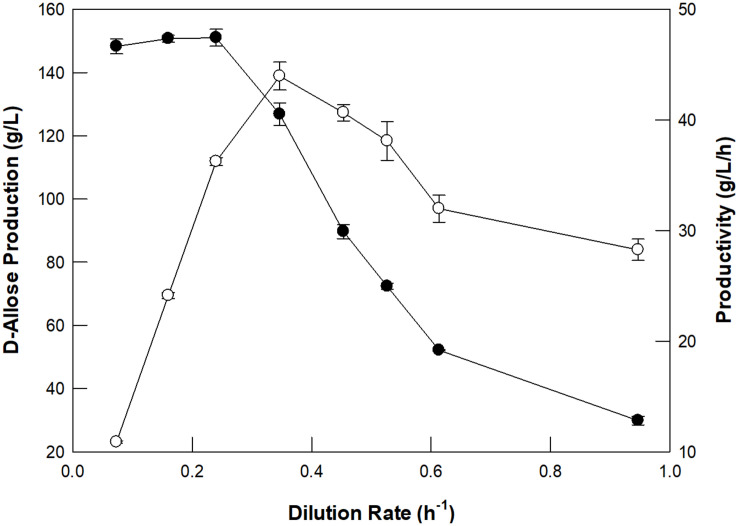
Effect of the dilution rate of D-allulose on D-allose production (closed circles) and productivity (open circles) using a packed bed reactor. The working volume of the reactor was 300 mL. A solution of 50 mM EPPS buffer (pH 8.0) containing 500 g/L D-allulose in the feeding reservoir was fed continuously into the reactor, and the effluent was allowed to flow out of the reactor to a reservoir using a peristaltic pump. The temperature was maintained at 70°C using a water circulator. Data represent the means of three experiments ± SD.

The optimum dilution rate for the continuous production of D-allose using a packed bed reactor was investigated at different dilution rates ranging from 0.07 to 0.95/h (Figure 4). From 0.07 to 0.35/h, the rate of production of D-allose increased, but the productivity and conversion yield began to decrease after 0.24/h, which is because the reaction time between the enzyme and the substrate shortens as the dilution rate increases. Thus, the optimal dilution rate for D-allose production was estimated to be 0.24/h. The optimum dilution rate for L-rhamnulose production from L-rhamnose using GI was reported to be 0.6/h (Kim et al., 2018). Although GI showed higher specific activity for D-allose than L-rhamnose, it was optimal at a lower dilution rate for D-allose than L-rhanmnose. This is presumably because the concentration of D-allose (500 g/L) used in the reaction was higher than that of L-rhamnose (300 g/L).

### Continuous D-Allose Production in a Packed Bed Reactor

**FIGURE 5:**
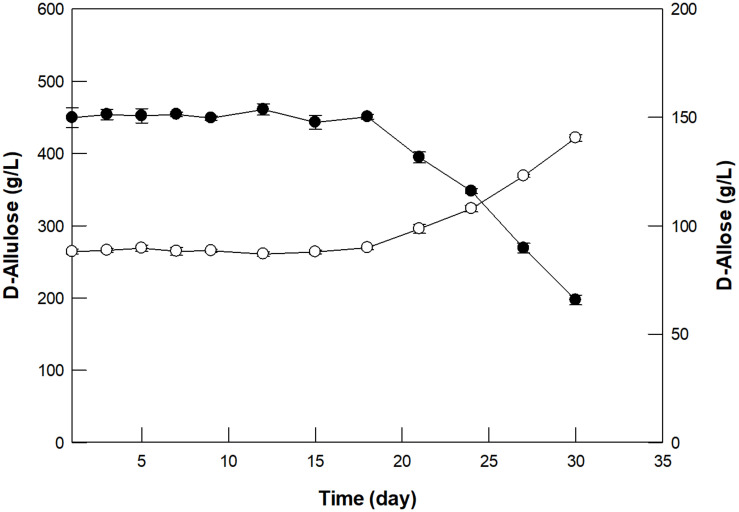
Continuous production of D-allose (closed circles) from D-allulose (open circles) using immobilized GI in a packed bed reactor. A solution of 50 mM EPPS buffer (pH 8.0) containing 500 g/L D-allulose was fed continuously into the reactor, and the effluent was allowed to flow out of the reactor at a dilution rate of 0.24/h. The reaction was performed at 60°C for 30 days. Data represent the means of three experiments ± SD.

GI was reused for 30 cycles with 500 g/L D-allulose under batch reaction for 4 h (Supplementary Figure 7). In the first batch reaction, GI produced 150 g/L D-allulose with a productivity of 37.5 g/L/h, which was about 1. 4-, 17. 9-, and 7,500-fold higher than those by free enzymes such as D-ribose-5-phosphate isomerase from *C. thermocellum* (Park et al., 2007b), D-galactose-6-phosphate isomerase from *L. lactis* (Park et al., 2007a), and glucose-6-phosphate isomerase from *P. furiosus* (Yoon et al., 2009), respectively. However, the productivity by GI was 2.1-fold lower than that of 79.6 by L-rhamnose isomerase from *C. stercorarium* (Seo et al., 2018). Nevertheless, GI exhibited more than 95 and 65% residual activity for 20 and 30 cycles, respectively, indicating the possibility of continued use of GI in continuous D-allose production. Based on the above experiments, the optimal conditions for D-allose production from D-allulose using GI in a 300-mL packed bed reactor were determined to be pH 8.0, 60°C, 500 g/L D-allulose, and a dilution rate of 0.24/h. Using these conditions, D-allose was produced continuously in the packed bed reactor for 30 days, a period within half-life, taking into account the thermostability at 60°C (Figure 5). An average of 150 g/L D-allose was produced from 500 g/L D-allulose substrate within 20 days, with a total of 5.18 kg D-allose, a productivity of 36 g/L/h, and a conversion yield of 30%. The D-allose concentration was reduced to approximately 43.9% after 30 days. In a previous report, immobilized L-rhamnose isomerase from *P. stutzeri* continuously produced 5.02 kg of D-allose from 16.6 kg of D-allulose over 30 days, with a conversion yield of 30% (Morimoto et al., 2006), which indicates about 1.5-fold lower productivity than that by GI. Therefore, continuous production of D-allose by GI can be a good alternative for industrial applications.

## Conclusion

Rare sugars are attracting attention as new functional materials because of their various biological properties. The rare sugars D-tagatose and D-allulose have already been industrialized, but D-allose has not. The rare sugar D-allose has approximately 80% of the sweetness of common sugar, but without the calories, and it exhibits several beneficial biological properties. For industrialization research and application purposes, the mass production of D-allose must be supported. However, there are no continuous production systems in place for the commercial synthesis of D-allose. In this study, we demonstrated that commercial food-grade D-glucose isomerase (Sweetzyme IT) converts D-allulose to D-allose by NMR analysis, investigated substrate specificity of the enzyme. For the continuous production of D-allose from D-allulose in a packed bed reactor, optimum reaction conditions such as pH, temperature, substrate concentration, and dilution rate were determined to be pH 8.0, 60°C, 500 g/L D-allulose, and 0.24/h, respectively. Under the optimum condition, 150 g/L of D-allose was produced from 500 g/L D-allulose, with a productivity of 36 g/L/h and a conversion yield of 30% within 20 days. This is a new approach for D-allose production, and to the best of our knowledge, this is the first report describing the continuous production of D-allose using a commercial enzyme. These results can be helpful for the industrial production of D-allose.

## Data Availability Statement

The original contributions presented in the study are included in the article/Supplementary Material, further inquiries can be directed to the corresponding author/s.

## Author Contributions

MC, K-CS, and Y-SK developed the concept and designed the manuscript. MC, K-CS, and DK provided key information and helped revise the manuscript. DK, B-JK, C-SP, and S-JY provided important intellectual support about experiment data. MC, K-CS, DK, B-JK, C-SP, S-JY, and Y-SK drafted the manuscript. All authors participated in writing and giving feedback on the manuscript and read and approved the final manuscript.

## Conflict of Interest

B-JK was employed by the company DAESANG Corporation. The remaining authors declare that the research was conducted in the absence of any commercial or financial relationships that could be construed as a potential conflict of interest.
