# Supplementary material for: Production of D-Allose From D-Allulose Using Commercial Immobilized Glucose Isomerase
Source: Front Bioeng Biotechnol. 2021 Jul 15;9:681253. doi: 10.3389/fbioe.2021.681253 (PMC8320891; doi:10.3389/fbioe.2021.681253)
Supplement: Supplementary file 1 [file Data_Sheet_1.docx]

**Production of D-allose from D-allulose using commercial immobilized glucose isomerase**

**Mi Na Choi^1†^, Kyung-Chul Shin^2†^, Dae Wook Kim^1^, Baek-Joong Kim^3^, Chang-Su Park^4^, Soo-Jin Yeom^5^, and** **Yeong-Su Kim^1*^**

^1^ Wild Plants Industrialization Research Division, Baekdudaegan National Arboretum, Bonghwa 36209, Republic of Korea; [mnchoi1022@kiam.or.kr](mailto:mnchoi1022@kiam.or.kr) (M.N.C.), [dwking@kiam.or.kr](mailto:dwking@kiam.or.kr) (D.W.K.)

^2^ Department of Integrative Bioscience and Biotechnology, Konkuk University, Seoul 05029, Republic of Korea; hidex2@konkuk.ac.kr

^3^ Starch & Sweetener Research Dept., Ingredient R&D center, DAESANG Corporation, Icheon 17384, Republic of Korea; [100-ing@daesang.com](mailto:100-ing@daesang.com)

^4^ Department of Food Science and Technology, Daegu Catholic University, Gyeongsan 38430, Republic of Korea; [parkcs@cu.ac.kr](mailto:parkcs@cu.ac.kr)

^5^ School of Biological Sciences and Technology, Chonnam National University, Gwangju 61186, Republic of Korea; [soojin258@chonnam.ac.kr](mailto:soojin258@chonnam.ac.kr)

**^†^**These authors contributed equally to this work and share first authorship.

*** Correspondence:**Yeong-Su Kim
[yskim@kiam.or.kr](mailto:yskim@kiam.or.kr)

**Supplementary Materials**


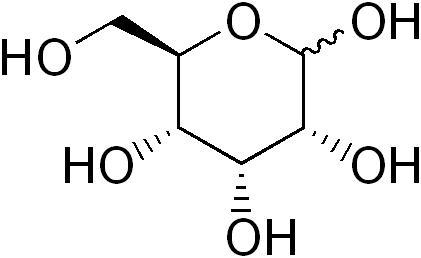


*d-Allose* : ^1^H NMR (800 MHz, D_2_O) *δ* 4.79 (d, *J* = 8.3, 1H), 4.08 (t, *J* = 2.9, 1H), 3.79 (dd, *J* = 12.2, 2.0, 1H), 3.69 (ddd, *J* = 10.1, 6.1, 2.3, 1H), 3.61 (dd, *J* = 12.2, 5.9, 1H), 3.54 (dd, *J* = 10.1, 3.0, 1H), 3.32 (dd, *J* = 8.3, 3.1, 1H). ^13^C NMR (200 MHz, D_2_O) *δ* 93.36, 73.55, 71.20, 71.11, 66.75, 61.12.

**Supplementary Figure 1**. ^1^H-NMR spectrum of allose (D_2_O, 800 MHz).

**Supplementary Figure 2**. ^13^C-NMR spectrum of allose (D_2_O, 200 MHz).

**Supplementary Figure 3**. HSQC-DEPT spectrum of allose.

**Supplementary Figure 4**. HMBC spectrum of allose.

**Supplementary Figure 5**. ^1^H-^1^H COSY spectrum of allose.

**A**

**B**

**Supplementary Figure 6**. The effect of pH and temperature on the production of d-allose from d-allulose by GI activity. (A) Effect of pH. The reactions were carried out with 10 g/L d-allulose in 50 mM buffers using acetate buffer (pH 4.0–5.5), MES buffer (pH 5.5–6.0), HEPES buffer (pH 6.0–8.0), and EPPS buffer (pH 8.0–9.0) at 60°C for 30 min. (B) Effect of temperature. The reactions were carried out with 10 g/L d-allulose in 50 mM EPPS buffer (pH 8.0) at 40–90 °C for 30 min. Data represent the means of three experiments ± standard deviation.

**Supplementary Figure 7**. Operational stability of the biocatalyst GI under batch reaction. Each reaction was performed in 50 mM EPPS buffer (pH 8.0) containing 500 g/L d-allulose at 60℃ for 4 h.
